# A new twist on graphene: an interview with Pablo Jarillo-Herrero and Allan MacDonald

**DOI:** 10.1093/nsr/nwac005

**Published:** 2022-01-13

**Authors:** Philip Ball

**Affiliations:** NSR from London

## Abstract

Graphene is the building block of graphite, made of carbon atoms bonded into sheets of hexagonal rings just a single atom thick. Although such isolated sheets had been predicted for many decades to exist, and had been grown on other surfaces, interest in this material exploded after the discovery in 2004 that single sheets could be made easily and cheaply by separating them mechanically from graphite flakes (a process called exfoliation). Although graphene is often advertised as a ‘wonder material’—electronically conducting, transparent and extremely strong and flexible—much of the interest in it is more fundamental. As a 2D conductor, graphene shows unusual electronic and magnetic properties that enable the study of quantum-mechanical effects of confinement and of correlations between electron motions—some of which might find applications in electronic devices. The excitement of this discovery was reflected in the award of the 2010 Nobel Prize in Physics to two pioneers in the field: Andre Geim and Konstantin Novoselov of the University of Manchester in the UK.

This rich behavior is broadened still further when two graphene sheets are brought close enough to interact with one another. In particular, the electronic properties may then depend on the relative orientation of the sheets: how aligned the two ‘honeycomb’ lattices are. Two grids superimposed on one another may create ‘superlattices’: regularities at larger scales than the grid spacing, due to registry (commensurability) between the two at certain angles. This so-called moiré effect is sometimes evident for two closely spaced grid-like fences seen from afar. Experimentally exploring the electronic properties of such ‘twisted bilayer graphene’ requires an ability to precisely control the position and orientation of the two sheets. These phenomena are now recognized as generic to other 2D materials, such as hexagonal sheets of boron nitride. They have revealed a fertile playground for condensed-matter physics. In particular, striking electronic properties appear at certain ‘magic-angle’ twists of the layers.

NSR spoke to two of the leading experts in the study of magic-angle twisted bilayer graphene (MATBG): experimentalist Pablo Jarillo-Herrero of the Massachusetts Institute of Technology and theorist Allan MacDonald of the University of Texas at Austin.

## THE DISCOVERY OF MAGIC ANGLES

***NSR:*** How did the discovery of unusual electronic behavior in twisted bilayer graphene came about? Were such effects predicted before they were observed?

***PJ-H:*** Many theory groups started to work on twisted bilayer graphene from around 2007. In late 2009, the group of Eva Andrei reported an investigation of twisted bilayer graphene using scanning tunneling microscopy (STM) [G. Li *et al.*, *Nat Phys* 2010; **6**: 109]. They observed peaks in their data that they interpreted as arising from features in the electronic structure known as Van Hove singularities [where the density of electronic states diverges]—which seemed to change with the twist angle. In particular, the separation between the peaks seemed to extrapolate to zero for a twist angle of ∼1.16°. Around the same time, two theory groups worked on the theory of twisted bilayer graphene at very small angles: Eric Suárez Morell and collaborators in Chile [E. S. Morell *et al.*, *Phys Rev B* 2010; **82**: 121407] and Rafi Bistritzer and Allan MacDonald in Texas [R. Bistritzer and A. MacDonald, *Proc Natl Acad Sci USA* 2011; **108**: 12233]. Both groups predicted the existence of ‘flat’ electronic bands in twisted bilayer graphene at an angle of 1.1–1.5°. Bistritzer and MacDonald coined the term ‘magic angle’: the angle for which the velocity of electrons at the Fermi level [the energy level electrons will occupy at zero temperature] goes to zero.

**Figure 1:**
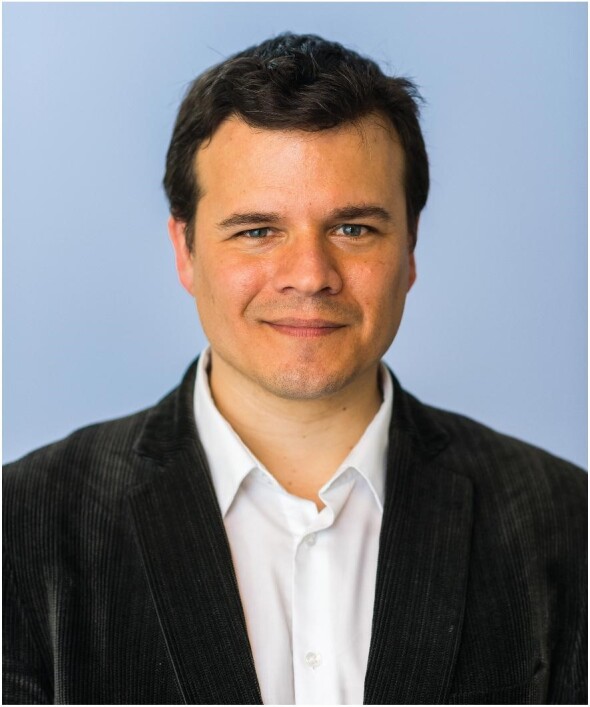
Experimentalist Pablo Jarillo-Herrero's group was the first to fabricate magic-angle graphene materials (*courtesy of Prof. Pablo Jarillo-Herrero*).

**Figure 2:**
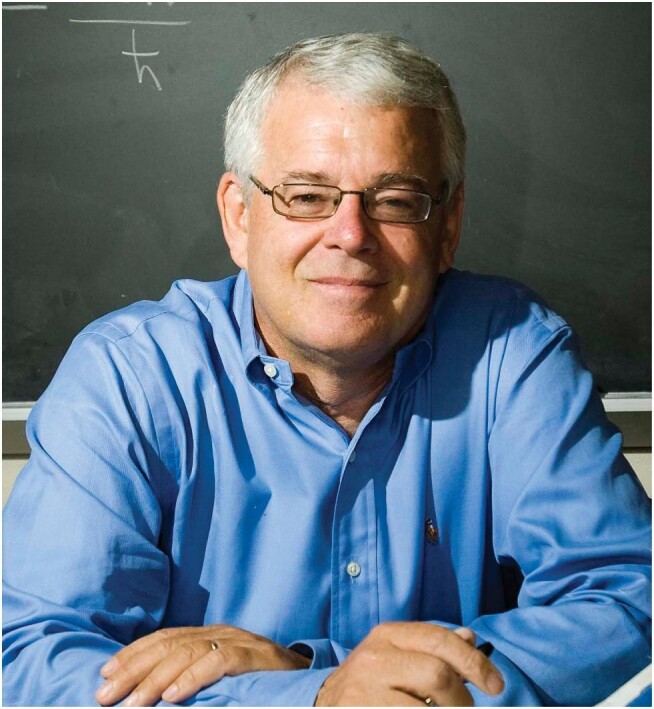
Allan MacDonald was one of the first theorists who predicted the special properties of magic-angle graphene (*courtesy of Prof. Allan MacDonald*).

My own group started to work on twisted bilayer graphene around 2010. In 2016, we showed that twisted bilayer graphene with a 1.8° twist angle already exhibited an interesting modification of the electronic structure: the velocity of the electrons at the Fermi level was substantially reduced, in agreement with theoretical calculations [Y. Cao *et al.*, *Phys Rev Lett* 2016; **117**: 116804]. This is what motivated us to continue working on reducing the twist angle and to investigate devices with twist angles very close to 1.1°. In 2017, we managed to fabricate several devices with twist angles in the range of 1–1.2°, and in these devices we made two unexpected discoveries. First, MATBG can become an insulator due to correlation effects when the charge density is tuned to a certain integer number of electrons or holes per moiré unit cell [Y. Cao *et al*., *Nature* 2018; **556**: 80]. Second, if you dope the material to add a bit of extra charge density, then MATBG becomes an electrically tunable superconductor with one of the highest ratios of critical temperature to Fermi temperature [Y. Cao *et al.*, *Nature* 2018; **556**: 43]. These characteristics are very reminiscent of other strongly correlated quantum materials and superconductors. The fact that we could obtain these results in such a seemingly simple system, just two layers of graphene rotated to a precise angle, and with such a degree of tunability, is one of the key reasons why our discovery attracted a lot of attention. These discoveries were totally unexpected and not predicted theoretically.

***AM:*** My understanding of the history—beyond what is in the publication record—comes from Eva Andrei [https://arxiv.org/pdf/2008.08129.pdf]. Eva was the first person to measure interesting changes in electronic structure, seeing features in STM density-of-states measurements on bilayer flakes that accidentally had moiré effects. Eva told me that the observation came first, and motivated the theory by Antonio Castro-Neto and João Lopes dos Santos.

My interest in graphene moiré electronic structure started from a conversation I had with Ed Conrad at Georgia Tech, who showed me some data from angle-resolved photoemission spectroscopy that I did not understand. As my postdoc Rafi Bistritzer and I built up our picture, we discovered that our calculations predicted that the velocity of graphene electrons would fall to zero at a discrete set of twist angles—we called them magic angles. The largest magic angle was ∼1°. This was a complete surprise to us, and we immediately recognized that it implied a rich platform for strong correlated-electron physics. Sometime later, we noticed that a group from Chile had independently obtained at least a glimmer of the magic-angle physics. At this time, we had no idea whether or not experimentalists would be able to fabricate samples with controlled twist angles to see this physics. My colleague Emanuel Tutuc did a lot of work in this direction, some of which helped to inform Pablo's parallel efforts.

***NSR:*** What induced you to study this system? It seems now that it offers a playground for looking at correlated-electron phenomena in a controlled way—but was that the expectation, or did the discovery come as a surprise?

***PJ-H:*** Originally, my motivation to study twisted bilayer graphene was the intuition that this ‘new knob’ in condensed-matter physics, namely the possibility to change the twist angle, had to yield interesting physics. In condensed-matter physics, the systems are typically quite complex, and when one explores unchartered territory, surprises often arise. In the specific case of magic-angle graphene, my motivation was to find interesting correlated insulator states that I though may appear when one would shift the Fermi energy in graphene to the Van Hove singularities. [NSR: New electronic phases such as superconductivity have been previously observed when the Fermi energy is close to such singularities.] We did find insulators—but to our surprise they were of a different type, where the insulating behavior occurs for an integer number of electrons per moiré unit cell, rather than because of the Van Hove singularities. This was a very nice surprise. An even bigger surprise was the discovery of superconductivity, which no one had predicted.

The magic-angle effect is a sort of ‘resonant’ condition … [where] it is as ‘easy’ for the electrons to go through one graphene layer as it is for them to ‘tunnel’ to the other graphene layer.

—Pablo Jarillo-Herrero

***AM:*** Our original theoretical finding of magic-angle effects was not expected on the basis of earlier experiments, and we had a lot of problems getting our work published because the referees thought that the result must be incorrect. Around this time, I was elected to the US National Academy of Sciences, which allows new members to publish a lightly reviewed inaugural article in the journal *PNAS.* I decided to give up on a long fight with referees and just publish our findings that way.

Following that paper, I tried to find other instances where interesting moiré superlattice physics would be observable. I proposed the possibility of realizing topological exciton bands [F. Wu *et al.*, *Phys Rev Lett* 2017; **118**: 147401] and a number of other proposals related to optical properties. I also proposed that layered transition-metal dichalcogenide (TMD) moiré systems would yield quite different physics compared to graphene multilayers. This part of the moiré field has now really taken off experimentally.

## A PLAYGROUND FOR NEW PHYSICS

***NSR:*** There seems to be a wide range of electronic states produced from these graphene systems, from insulators to superconductors and magnetic systems. What is the underlying physics that produces such a menagerie of states, and what are the key factors that determine the properties?

***PJ-H:*** Well, we are still trying to fully understand these systems. But your basic observation is true: MATBG, and now several other moiré systems, exhibit an incredibly rich set of correlated behaviors. The origin seems to be in the fact that these systems have narrow electronic bands (meaning the electrons have very little kinetic energy) and hence the interaction energy [due to electrostatic repulsion] between the electrons plays a dominant role. Once you have strong interactions between the electrons, then many possible many-body ground states (for example, superconductivity, correlated insulators, magnetism, etc.) become possible. We are seeing all of these because moiré systems are highly tunable.

***AM:*** There are many analogies between strong correlations in graphene multilayers and strong correlations in quantum Hall physics. This connection was clarified by work of Eslam Khalaf, Ashvin Vishwanath and collaborators at Harvard, Mike Zaletel and others. Ultimately it related to topological properties of the electronic bands. At the same time, these systems have an aspect of the quasi-2D Hubbard model [one of the first and simplest lattice models of strongly correlated electrons]. MATBG seems to be a marriage of the quantum Hall effect [which arises in 2D systems] and high-temperature superconductivity—it's an amazing system.

***NSR:*** Can you explain the magic-angle effect? What makes certain relative orientations of the graphene sheets ‘special’?

It's a whole new paradigm for making artificial tunable crystals, and we’re still just scratching the surface. We’ll see what happens—that's what makes science fun.

—Allan MacDonald

***PJ-H:*** The magic-angle effect is a sort of ‘resonant’ condition. One can think of the magic angle as the angle for which the electronic structure is such that it is as ‘easy’ for the electrons to go through one graphene layer as it is for them to ‘tunnel’ to the other graphene layer. In much simpler terms, an analogy for why electrons in MATBG display such varied behavior is this: when electrons have lots of kinetic energy (that is, they move very fast), they barely have time to interact. But in MATBG, the electrons are very slow, and hence they have lots of opportunity to interact with each other as they pass each other by.

***NSR:*** The interplay of insulating and superconducting behavior in this system seems to mirror that seen in the copper-oxide high-temperature superconductors. Is similar physics at work? Does this behavior in fact help us to understand the origin of superconductivity in such materials?

***PJ-H:*** There are indeed many similarities between the phase diagrams of MATBG and cuprate superconductors, but there are also many differences too. For example, the symmetries of the lattices and the topological properties of the electronic structures are very different. Also, the electrons in the cuprates have degenerate spin [all the same], whereas the spin states for MATBG are somewhat richer. So we do not know yet whether understanding MATBG will help us to understand the origin of superconductivity in cuprates. My intuition is that it will, but it's too early to tell.

***AM:*** We do not yet have completely confident answers to these questions, but we are making progress. There are many similarities between high-temperature superconductors and MATBG systems—the proximity to magnetic order and to Fermi-surface reconstructions is most intriguing. In my opinion, there is every reason to expect that we will continue to make progress in understanding MATBG superconductivity by performing new experiments and testing theoretically proposed scenarios, and that this progress will have implications for understanding high-temperature superconductivity. The possibility of tuning the charge-carrier density *in situ*, and the possibility of adjusting the system properties in other ways (for example, by varying the distance to gates, the dielectric environment and the in-plane magnetic field) is an important advantage for MATBG.

***NSR:*** What is the role of dimensionality here? Are these behaviors dependent on the fact that this is a (quasi-)2D system? And in that regard, does this behavior connect with the work on low-dimensional quantum many-body problems such as the quantum Hall effect (QHE)?

***PJ-H:*** Dimensionality is quite important for various reasons. Among them: MATBG is highly tunable electrically because of the 2D geometry; the electronic structure (for example, the density of states) depends on dimensionality; and interaction effects can be also strongly dependent on dimensionality—for example, screening by electrons is very different in 1D, 2D and 3D. Regarding quantum Hall physics, there is a deep connection in that both the QHE and the electronic bands in MATBG (and several other related moiré systems) are topological in nature. That's why the latter can show interesting quantum Hall effects, even at zero magnetic field [unlike the standard QHE].

***AM:*** Electronic correlations tend to be stronger in lower-dimensional systems and have more scope to yield surprising many-electron states, including fractional quantum Hall effect (FQHE) systems, MATBG, bilayer and trilayer graphene. The topological picture of the QHE provides a link between MATBG and FQHE physics. One experimental proof of this connection is the common appearance of anomalous quantum Hall states (that is, QHE without a magnetic field) in MATBG.

## CHALLENGES, APPLICATIONS AND OPPORTUNITIES

***NSR:*** How does one study these systems experimentally? Is it now routine to make good-quality single-layer graphene? How do you control the relative orientation of the sheets?

***PJ-H:*** It is very standard to obtain very-high-quality single-layer graphene, by mechanical exfoliation of graphite for example. Thousands of groups around the world can do this. What is a lot more tricky is to be able to stack on top of each other two graphene sheets with a very well controlled angle of rotation, and that's even harder for small angles such as the magic angle of 1.1°. There are only ∼15 groups around the world that can currently fabricate MATBG, but more are joining all the time. It's something that can be easily learned if someone shows you. Before the pandemic, we had plenty of groups come to MIT and learn about it, and those groups now have reproduced and extended many of our results.

***AM:*** It is amazing what has been accomplished already. If it were possible to develop techniques to control twist angles even more finely, and make the twist angles even more uniform, it would speed up progress in the field.

***NSR:*** What are the key questions that still remain to be explored in these systems? What are you personally most eager to study now?

***PJ-H:*** There are many key questions remaining to be explored. Perhaps one of the most important is the exact mechanism for superconductivity and the symmetry of the order parameter. Right now, experiments and theory seem to point towards a very unconventional origin for the superconductivity (though not

There are only ∼15 groups around the world that can currently fabricate MATBG, but more are joining all the time.

—Pablo Jarillo-Herrero

everybody agrees, and some people still think MATBG could be a [standard] electron–phonon-mediated superconductor in a very unusual parameter regime). But we still need to study this in more detail. I’m personally very eager to discover and study new moiré systems, new superconductors and their correlated and topological behavior. I think we have barely scratched the surface of the many hundreds of possible moiré systems we can build, with very different constituents, geometries and complexity.

***AM:*** I think that it is important to nail down the origin of superconductivity in MATBG. I am actively working on this question.

One important hope is that we will be able to realize fractional anomalous quantum Hall systems (also known as fractional Chern insulators) in MATBG or in TMD moirés that show the quantum anomalous Hall effect. Given the flexibility of the moiré superlattices, there is a good chance that favorable conditions could be discovered or engineered. FQH states are one of the possible hosts for topological quantum computing too.

***NSR:*** There seem to be many potential degrees of freedom to explore in such systems. For example, there is now interest in going beyond bilayer systems to trilayers—what is predicted and/or observed there? What about using hetero-bilayers, such as with boron nitride or other 2D materials?

***AM:*** I am very interested in identifying new layered materials that can host new classes of moiré superlattices—each case will bring its own universe of new physics. Already just with TMD and twisted graphene moirés, we have examples of itinerant [mobile] electron ferromagnetism—but with quite low ordering temperatures. It's interesting to think about how ordering temperatures can be increased and what the ultimate limits are. Because the moiré superlattice systems can be tuned in so many ways, there is some scope for optimism. It's a whole new paradigm for making artificial tunable crystals, and we’re still just scratching the surface. We’ll see what happens—that's what makes science fun.

***PJ-H:*** Indeed, the possibilities are pretty much endless. Just earlier this year, two independent groups (Philip Kim's group and my group) discovered superconductivity in magic-angle twisted trilayer graphene (MATTG). The magic angle turns out to be a bit different (∼1.6°), which was theoretically predicted a couple of years ago, so we knew where to look. The superconductivity in MATTG turns out to be even more interesting than in MATBG, because it is stronger and much more tunable. Using hetero-bilayers can indeed add many things, and the discovery of the quantum anomalous Hall effect (QAHE) in MATBG aligned with boron nitride was one of the earliest examples.

***NSR:*** More generally, MATBG systems exemplify an explosion of interest in the past two decades in strongly correlated electrons, which has yielded the discovery of a vast zoo of quantum materials, such as topological insulators, Majorana zero modes, Weyl semimetals and so on. What has triggered the explosion of interest? Is there any emerging sense of a unified view of the quantum electronic phases of matter, or are we still very much in the phase of discovery and surprise?

***PJ-H:*** Condensed-matter physics saw two ‘revolutions’ in the 1980s, with the discovery of the integer/FQH effects (which brought topology into the field) and with the discovery of high-temperature superconductivity (which brought strong correlations to the forefront). Since then, these two communities (working on topological and strongly correlated systems) did not interact very closely, because the systems were quite different. Then in the 2000s, three major disruptions occurred: the discovery of graphene and 2D crystalline materials; the theoretical prediction and, soon after, the experimental discovery of topological insulators; and the discovery of a second family of high-temperature superconductors, the iron pnictides. Yet still these communities remained largely separate. MATBG has merged these three communities, because it has characteristics of all of them. This topic of ‘moiré quantum matter’ has stimulated very interesting discussions among all of these people.

***AM:*** In my view, we are still very much in a phase of discovery and surprise, but I am very optimistic that these new classes of strongly correlated systems will lead to a broader and deeper view of strong-electron-correlation physics.

***NSR:*** Are there any likely practical applications of such systems? In particular, does there seem any likelihood that MATBG might find its way into device technology?

***PJ-H:*** This is always very hard to predict. For now, my group and the overall community are motivated by the beautiful fundamental physics that we can explore in these systems. Having said that, MATBG is an electrically tunable superconductor or, in engineering terms, a superconducting field-effect transistor, so one could easily imagine many applications for it, if the community can manage to fabricate MATBG on a large scale. Applications easy to imagine include tunable superconducting quantum bits, tunable quantum photodetectors and classical cryogenic computing.

***AM:*** Personally, I am very interested in identifying potential applications—for optical properties perhaps, or for spintronics [information processing using spins]. Interfaces with TMDs may prove useful in adjusting spin–orbit interaction strengths—something that will be key for spintronics.

***NSR:*** What is your impression of the research in this area in China?

***PJ-H:*** There has been lots of interest in China from the point of view of theoretical physics. In terms of experimental work, China currently has only a few groups with the nanofabrication experience to produce high-quality moiré quantum systems. Those groups (the most renowned of which is that of Yuanbo Zhang (张远波) at Fudan University) are producing very good work. Given the recent rapid evolution of scientific research in China, I expect many more experimental groups will start working on this subject in the coming years.

My former student Yuan Cao (曹原) is a very remarkable scientist in many respects. He is very smart, hard-working, creative and effective. He was not only the first author in the two discovery papers I mentioned above, and hence a young leader in the field, but has continued to make outstanding contributions to the field since then. He has received several awards, even at his young age, including the McMillan Award (the most prestigious award for young condensed-matter physicists) and very recently the International Sackler Prize in Physics. I consider myself extremely fortunate to have worked with him. I think I have learned, at the very least, as much from him as he may have learned from me. I believe he will be one of the leading scientists of his generation.

***AM:*** Fengcheng Wu (吴冯成), a former student in my group who did important early work on TMD moirés, including optical and electronic properties, and on MATBG superconductivity, is now a professor at Wuhan University and is a leading researcher in the field. Wang Yao (姚望) at Hong Kong University is a leading theoretical researcher on optical properties of TMD moirés. The QAHE was first observed at Tsinghua University in magnetic topological insulators. MATBG has provided a second example, with interesting similarities and differences.

Most of the time, the new understanding is just a detail that is already understood by someone—but every now and again it will be something truly new.

—Allan MacDonald

***NSR:*** What or who are your key sources of inspiration for this work? And what advice would you give to young researchers entering the field?

***PJ-H:*** There are many colleagues whom I find very creative and who have inspired my group's approach to experimental condensed-matter physics. These include Paul McEuen (Cornell), Andre Geim (Manchester) and Amir Yacoby (Harvard). And, of course, my PhD advisor Leo Kouwenhoven at Delft and my postdoc advisor Philip Kim at Harvard had a big influence in my formation. To a young researcher, I’d say: be adventurous and take risks. Try to follow your interests and don’t let others put a ceiling on your ambitions.

***AM:*** I’ve been doing this a long time now. I have developed a great appreciation for the ability of experiment to surprise. My approach to doing fundamental theory in materials science is to try to identify exciting new phenomena that are likely to be observable experimentally if someone would just look. My intuition is based very much on known experimental results and by thinking about why different types of theoretical approximations are successful—or unsuccessful—in describing nature. Trying to deepen theoretical understanding of phenomena that have been observed but remain mysterious is nearly as much fun.

I would advise young researchers to develop their own independent way of thinking about their scientific area. Whenever you encounter something that you do not understand, dig deeper until you do. Most of the time, the new understanding is just a detail that is already understood by someone—but every now and again it will be something truly new.

## Notes

Philip Ball writes for NSR from London.

